# Understanding Agri-Food Traceability System User Intention in Respond to COVID-19 Pandemic: The Comparisons of Three Models

**DOI:** 10.3390/ijerph19031371

**Published:** 2022-01-26

**Authors:** Yafen Tseng, Beyfen Lee, Chingi Chen, Wang He

**Affiliations:** 1 Digital Design and Information Management, Chung Hwa University of Medical Technology, Tainan 71703, Taiwan; 2 Department of Hospitality Management, Chung Hwa University of Medical Technology, Tainan 71703, Taiwan; 3 Department of Health Care Administration, Chung Hwa University of Medical Technology, Tainan 71703, Taiwan; 4 School of International Business, Jiangxi University of Finance and Economics, Nanchang 330013, China; hewang2004@126.com

**Keywords:** COVID-19, continuance intention, traceability system, technology acceptance model, information systems success model, expectation confirmation model

## Abstract

Scientists believed the outbreak of COVID-19 could be linked to the consumption of wild animals, so food safety and hygiene have become the top concerns of the public. An agri-food traceability system becomes very important in this context because it can help the government to trace back the entire production and delivery process in case of food safety concerns. The traceability system is a complicated digitalized system because it integrates information and logistics systems. Previous studies used the technology acceptance model (TAM), information systems (IS) success model, expectation confirmation model (ECM), or extended model to explain the continuance intention of traceability system users. Very little literature can be found integrating two different models to explain user intention, not to mention comparing three models in one research context. This study proposed the technology acceptance model (TAM), technology acceptance model-information systems (TAM-IS) success, and technology acceptance model-expectation confirmation model (TAM-ECM) integrated models to evaluate the most appropriate model to explain agri-food traceability system during the COVID-19 pandemic. A questionnaire was designed based on a literature review, and 197 agri-food traceability system users were sampled. The collected data were analyzed by partial least square (PLS) to understand the explanatory power and the differences between the three models. The results showed that: (1) the TAM model has a fair explanatory power of continuance intention (62.2%), but was recommended for its’ simplicity; (2) the TAM-IS success integrated model had the best predictive power of 78.3%; and (3) the system providers should raise users’ confirmation level, so their continuance intention could be reinforced through mediators, perceived value, and satisfaction. The above findings help to understand agri-food traceability system user intention, and provide theoretical and practical implications for system providers to refine their system design.

## 1. Introduction

Food plays an important role in human life, so food safety is one of the major concerns worldwide [1]. Food safety incidents, such as mad cow disease, alert consumers to be more concerned about the food they eat. Recently, due to the possibility that the COVID-19 virus is transmitted from wildlife to humans [2], consumers’ worries about food safety are enormously high. Therefore, people are willing to spend more money on safe food, i.e., traceable food with its provenance certified.

The main concerns when customers purchase food relate to the source and the hygiene of the food itself. To better understand the food quality, customers request clarity from food suppliers. Businesses should be capable to verify the content and source of their commodities to safeguard the customers in opposition to deception. In this situation, traceability and verification are essential instruments for assuring customers in conditions of food disclosure and protection, and in permitting manufacturers to obtain knowledge of their goods. Traceability allows the tracing of the supply of food at every point in the manufacturing chain, allowing the value-management methods, and reducing the manufacture of hazardous foods [3]. Food verification is the procedure through which food is analyzed to confirm if it conforms with the explanation included in its description [4]. Traceability and verification are essential elements of food protection, and correspond to basic parts of the food supply chain. A consistent validation and traceability structure can represent an important method for the safety of customers, decreasing the risk of individuals utilizing impure or tainted foods, and improving supplier management and procedure protection. Customers demonstrated inadequate understanding regarding the significance of validation and traceability of food [5,6], making the distribution of the ability and dependability of tracking techniques to improve individuals’ understanding of the position of food supervision in health security and the honesty of traceability knowledge important [7]. Food supply chains are vital to well-being. During the pandemic, countries need to maintain their daily operations. Investigators have examined the pandemic’s influence on the food business in remarkable detail [8,9,10,11]. The COVID-19 pandemic has interrupted food supply chains globally [12]. Various regulations and guidelines have been established and published in government and private segments to strengthen the food business, and avoid contamination from propagating. The Chinese State Council additionally issued a bill to avoid the pandemic contamination threats in food chains [13].

In many countries, the food and agriculture sectors are considered to be a major production and supply chain in a nation. Complex food supply chains that lack transparency and traceability make it very difficult for the government to find the outbreak origins when food safety issues occur. Thus, relevant directives, laws, as well as standards and regulations were established to ensure the traceability of food products. Consequently, the development of traceability systems throughout the food supply chains is considered to be a solution that could resolve the impact of food crises fast. Food traceability systems have progressively become the consumers’ concern, the manufacturers’ marketing strategies, and governments’ policy initiatives for the following three reasons: firstly, consumers believe food with traceability is safer than those that cannot be traced. Secondly, a food manufacturer can price traceable food higher than those without traceability. Thirdly, the government believes it helps to trace back the food origin, and speeds up the processing time during food crisis outbursts. However, even with the support of the government, not all food traceability system deployment is successful [14]; the users’ continuance intention needs to be further explored, especially during the COVID-19 pandemic.

The technology acceptance model (TAM), information systems success model (IS success model), and expectation confirmation model (ECM) have been widely used in information systems studies to understand consumers’ behavior intention. Traceability systems involve information and logistic systems, so these models are applicable to the traceability systems. The purpose of this study is to understand the continuance intention of traceability system usage during the COVID-19 pandemic by comparing three different models, including TAM, TAM-IS success, and TAM-ECM integrated models. In this study, partial least squares (PLS) were used to analyze data collected by the agri-food traceability system users to evaluate the relationships among user perception (perceived ease of use and perceived value), system evaluation (system quality, information quality, and service quality), confirmation, satisfaction, user habits, and continuance intention. The advantages of the three models can be compared in explaining user continuance intention, and the model with the best explanatory power for the traceability system can be determined.

Unlike most of the studies, which adopted only one model as the theoretical foundation, this study integrates the TAM model, the IS success model, and the ECM. Three conceptual models are proposed in this study to explain consumers’ continuance intention to use the traceability system. Also, this study investigates the relationships among three models, including constructs such as perceived ease of use, perceived value, system quality, information quality, service quality, confirmation, satisfaction, and continuance intention. This study covers at least three major research gaps. First, it analyzes the inter-relationships between TAM and behavioral intention. Second, it examines the relationships between service quality, information quality, and system quality, with perceived ease of use and perceived value. Furthermore, it examines the relationships between perceived ease of use and perceived value with continuance intention. Third, it investigates the relationships of confirmation with perceived value and perceived ease of use. Additionally, it also examines the association between satisfaction and continuance intention. Moreover, this study analyzes the indirect mediation effects of the models presented in this study. Finally, this study uses “user habits” as a moderator for the relationships between perceived value and continuance intention, and satisfaction and continuance intention.

## 2. Literature Review

### 2.1. Traceability System

After the second mad cow disease crisis occurred in Europe in 1996, the European Union launched an agricultural traceability system to improve food safety. The prevalence of COVID-19 has made people more concerned about food safety because the origin of the disease might be from wildlife. Food traceability refers to the ability to track the flow of food or ingredients through specific stages of production, processing, and distribution [15]. When potential food safety or security issues are identified, traceability systems allow the implementation of corrective actions and pausing risks to public health. The use of food traceability systems could quickly isolate the polluted items from the supply chain, and prevent contaminated products from reaching consumers. When public food safety issues occur, government authorities interfere to avoid the potential number of illnesses or deaths of the public, and to diminish the damage on the markets. For tracing back to the food origin quickly, countries such as Australia, India, China, New Zealand, South Korea, Taiwan, Thailand, and the United States have developed agricultural traceability systems in past decades.

Besides the demand from the authorities, traceability systems could be motivated by economic incentives, in that manufacturers can differentiate their food products in the market based on their credibility [16]. Companies can attract consumer interest by showing the traceability of organic, non-GMO food, or point of origin, so the product price, customer satisfaction, and profit margin could be raised. To the consumers, food with traceability meets their expectations for food safety. Consumers are willing to pay a premium price for traceable food for the assurance of food safety [17,18,19]. Table 1 summarizes previous literature on traceability systems, which were based on different theories and dependent variables in different contexts. Many studies adopted TAM or its extensions as the theoretical foundation. However, empirical research grounded on the information systems success model or expectation confirmation model is rare.

### 2.2. Technology Acceptance Model (TAM)

**Figure 1:**
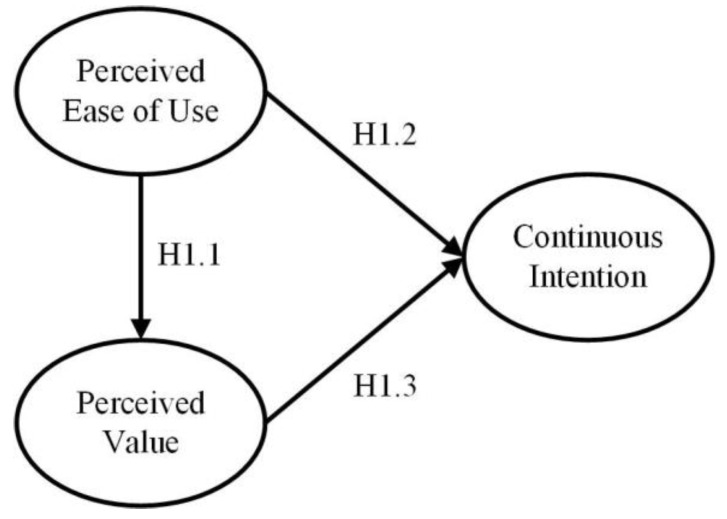
Research Model I (TAM).

The TAM has been widely adopted by scholars for its streamlined structure and strong explanatory power, and was first proposed by Davis [29] to explain the factors influencing the user acceptance of computer systems. The model contains only four constructs: user behavior is influenced by behavioral intention, which in turn is impacted by perceived ease of use and perceived usefulness. Davis [29] defined perceived usefulness as the subjective perception of users where they believe that using certain technologies can improve the performance of their work. Based on the “perceived value” defined by Zeithaml [30], this study defined perceived value as the overall assessment of the usefulness of the traceability system based on users’ perceptions of what they receive compared with what they give. According to the TAM, cognitive beliefs have effects on behavioral intention, so this study substituted the construct perceived usefulness with perceived value as Figure 1, and proposed the following hypotheses:

**Hypothesis** **1.1** **(H1.1).** *Perceived ease of use has a positive impact on perceived value.*

**Hypothesis** **1.2** **(H1.2).** *Perceived ease of use has a positive impact on behavioral intention.*

**Hypothesis** **1.3** **(H1.3).** *Perceived value has a positive impact on behavioral intention.*

### 2.3. TAM-IS Integrated Model

The information systems success model (IS success model), explaining the constitution of IS success, was developed by Delone and McLean [31], and improved a decade later. The updated IS success model [32,33,34] is considered to be one of the most influential theories in the field of information systems research because it explains the production, use, and net benefits of IS. Information, system, and service quality in the IS production phase will impact the consumers’ intention to use and their satisfaction, which will then affect individual or organizational productivity.

**Figure 2:**
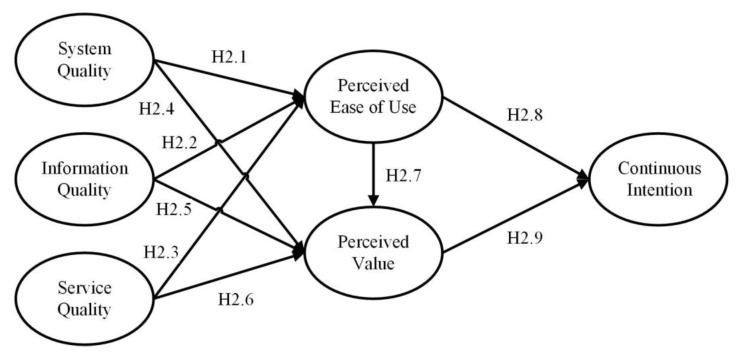
Research Model II (TAM-IS Success Integrated Model).

The TAM-IS integrated model (Figure 2) is the second proposed model in this study. This model concerned the users’ continuance intention of an agri-food traceability system. There are six variables in this model, including system quality, information quality, and service quality, which were derived from the IS success model as the predecessors of perceived ease of use and perceived value, and had a direct impact on continuance intention.

Mustapha and Obid [35] showed a direct positive relationship between online tax service quality and perceived ease of use. Bahari et al. [36] showed a similar result in their hotel website design studies. Li and Shang [37] suggested that e-government service quality affected perceived service value. Ali and Younes [38] asserted that information quality positively affected the perceived ease of use. Machdar [39] has a similar finding in her accounting class. Tsao et al. [40] indicated system quality and e-service quality both had a positive effect on the perceived value of consumers or sellers. In an e-commerce study, Putri and Pujani [41] proved the effects of system quality, information quality, and e-service quality on perceived value. Therefore, it is reasonable to infer similar concepts, and propose the following hypotheses:

**Hypothesis** **2.1** **(H2.1).** *System quality has a positive and significant effect on perceived ease of use.*

**Hypothesis** **2.2** **(H2.2).** *Information quality has a positive and significant effect on perceived ease of use.*

**Hypothesis** **2.3** **(H2.3).** *Service quality has a positive and significant effect on perceived ease of use.*

**Hypothesis** **2.4** **(H2.4).** *System quality has a positive and significant effect on perceived value.*

**Hypothesis** **2.5** **(H2.5).** *Information quality has a positive and significant effect on perceived value.*

**Hypothesis** **2.6** **(H2.6).** *Service quality has a positive and significant effect on perceived value.*

Sarmento and Mesquita [42] found that ease of use has a significant positive effect on perceived value. In the study of online exchanges [43], easy navigation of the C2C website was vital for consumers to perceive value from using the platform. Based on TAM, perceived ease of use had a direct impact on behavior intention. As to the relationship between perceived value and behavior intention, Jin and Lee [44] found that perceived value had a direct influence on behavioral intention in their water park research. Similar conclusions could be drawn in the studies of Meng et al. [45], Jen et al. [46], and Kamtarin [47]. Therefore, the following hypotheses are proposed:

**Hypothesis** **2.7** **(H2.7).** *Perceived ease of use has a positive and significant effect on perceived value.*

**Hypothesis** **2.8** **(H2.8).** *Perceived ease of use has a positive and significant effect on continuance intention.*

**Hypothesis** **2.9** **(H2.9).** *Perceived value has a positive and significant effect on continuance intention.*

**Hypothesis** **2.10** **(H2.10).** *There are mediation effects in this model.*

### 2.4. TAM-ECM Integrated Model

The expectation confirmation model (ECM), which evolved from the expectation confirmation theory [48], focuses on the comparison between consumers’ expectations before purchasing a product or service, and their performance in using the product or service to determine consumers’ satisfaction with the product. Because ECT showed good explanatory and predictive power in the traditional marketing field, scholars apply it to different types of information system products that established the ECM model [49]. The ECM model aims to understand the impact of confirmation and perceived value on continuance intention through satisfaction, and has been widely used in the various IS contexts, such as distance education [50,51,52], online services [53,54], and mobile apps [55,56]. Bhattacherjee [49] asserted IS users’ continuance decision is similar to consumers’ repurchase decision, so ECM is appropriate as the theoretical base for this study.

**Figure 3:**
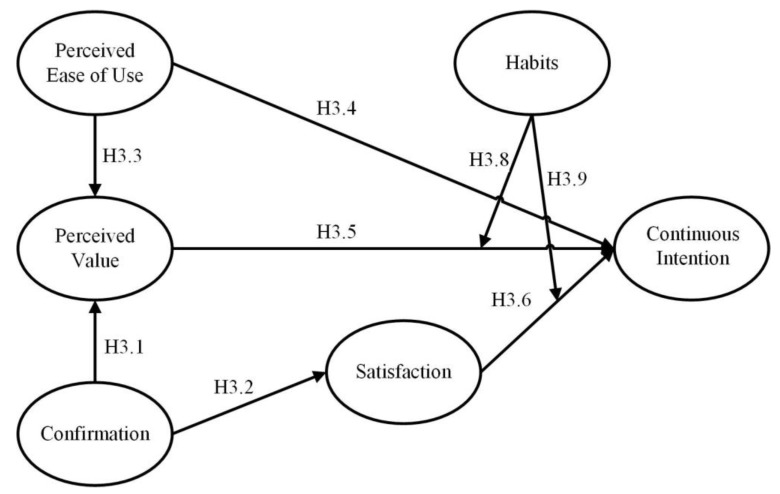
Research Model III (TAM-ECM Integrated Model).

The TAM-ECM integrated model is the third proposed model in this study, as shown in Figure 3. This model introduced the constructs perceived ease of use and perceived value from the TAM, and confirmation and satisfaction from the ECM. A study of paid mobile apps showed that confirmation affected both perceived value and satisfaction [57]. Therefore, this study proposes the following hypotheses:

**Hypothesis** **3.1** **(H3.1).** *Confirmation has a positive and significant effect on perceived value.*

**Hypothesis** **3.2** **(H3.2).** *Confirmation has a positive and significant effect on satisfaction.*

**Hypothesis** **3.3** **(H3.3).** *Perceived ease of use has a positive and significant effect on perceived value.*

**Hypothesis** **3.4** **(H3.4).** *Perceived ease of use has a positive and significant effect on continuance intention.*

**Hypothesis** **3.5** **(H3.5).** *Perceived value has a positive and significant effect on continuance intention.*

**Hypothesis** **3.6** **(H3.6).** *Satisfaction has a positive and significant effect on continuance intention.*

**Hypothesis** **3.7** **(H3.7).** *There are mediation effects in this model.*

Habits were defined as automatic behaviors without self-instruction [58]. Khalifa and Liu [59] asserted that habits might have effects on the determinants of continuance purchase intention. In the IS context, Limayem et al. [60] defined habit as the use of a particular IS that has become automatic in response to certain situations. Limayem and Cheung [61] believed habits are a moderator in IS continuance usage. Also, empirical studies have examined the moderating effect of habits on the relationship between perceived value and repeat purchase intention [62,63], and the relationship between satisfaction and repeat purchase intention [59,63,64]. Thus, habit is included in the TAM-ECM integrated model to test its moderating effects on the linkages between continuance intention purchase intention and its antecedents (i.e., perceived value, satisfaction). Thus, the following hypotheses were proposed:

**Hypothesis** **3.8** **(H3.8).** *User habits have a moderator effect on the relationship between perceived value and continuance intention.*

**Hypothesis** **3.9** **(H3.9).** *User habits have a moderator effect on the relationship between satisfaction and continuance intention.*

## 3. Research Method

Questionnaires were developed based on the literature review, then modified to fit the purpose of this study. A questionnaire that measures constructs in the TAM, including perceived ease of use, perceived value, and continuance intention to use, was based on the study of David [29]. A questionnaire to determine the constructs of TAM-IS, including system quality, information quality, service quality, perceived ease of use, perceived value, and continuance intention to use, was modified from the work of DeLone and McLean [31]. The questionnaire assessing the constructs of TAM-ECM, including perceived ease of use, perceived value, confirmation, satisfaction, usage, and continuance intention to use, was modified from the research of Bhattacherjee [49]. Questionnaire items to measure all constructs are shown in the Appendix A. A 7-point Likert scale was used for each question, where 1 signifies strongly disagree, and 7 represents strongly agree. User demographic information, such as gender, age, education, business scale, and income, was also collected.

Before the formal survey, 35 users were chosen for the pilot study, and survey items were revised to improve the quality of the questionnaire. This study explored the continuance intention of the agri-food traceability system. Participants were sampled in a traceability-system-related conference organized by Ganzhou City Fruit Industry Bureau during the COVID-19 period. The survey targeted users who have used the agri-food traceability system for over one year. A total of 245 paper questionnaires were distributed, and 207 were collected. Among them, 197 questionnaires were valid, meeting the requirement suggested by Anderson and Gerbing [65] that the minimum sample size should be at least 150 if the analysis method is structural equation modeling. Since the respondents were drawn from a conference, the sample may not distribute normally. Therefore, PLS was used to avoid the effects of data distortion, and to quantify the relationship among constructs. Confirmatory factor analysis was used to test the measurement model’s reliability and validity. After determining the fitness of the research model and the data, hypotheses tests were performed based on the analytical results of standardized factor loadings, path coefficients, and *p* values.

## 4. Analysis Result

### 4.1. Measurement Model

#### 4.1.1. Convergent Validity

To assess the convergent validity of the measurement items, Fornell and Larcker [66] proposed three indexes, including: (a) item reliability of each measure or square multiple correlations, (b) composite reliability of each construct, and (c) the average variance extracted (AVE). As Table 2 shows, all standardized factor loadings of questions are from 0.5 to 0.770, exceeding the 0.5 value recommended by Hair et al. [67]. This demonstrates all questions have convergent validity. All the composite reliability (CR) of the constructs ranging from 0.762 to 0.874 exceed the 0.7 value recommended by Nunnally and Bernstein [68], indicating all constructs have internal consistency. Lastly, all average variance extracted (AVE), ranging from 0.50 to 0.698, exceed the 0.5 value suggested by Hair, Anderson, Tatham, and Black [69], as well as Fornell and Larcker [66], showing all constructs have adequate convergent validity.

#### 4.1.2. Discriminant Validity

In Table 3, discriminant validity appears to be satisfactory for all constructs because the bold numbers in the diagonal direction are greater than the off-diagonal numbers (Fornell and Larcker [66]).

### 4.2. Structural Model Analysis

This study uses the maximum likelihood method to estimate the hypothesized relationships of the proposed model. Model fit indicators determine the degree to which the sample data fit the structural equation model (SEM). This study adopted a variety of criteria to determine the model fit, as recommended by Jackson et al. [70]. The model fit indicators in Table 4 satisfy most of the recommended levels, so the three proposed models have good model fits.

In order to indicate a good fit, the root mean square error of approximation (RMSEA) value should be less than 0.08 [71]. The goodness of fit index (GFI) and adjusted goodness of fit index (AGFI) values should have the standard upper bound of 1. Similarly, for the non-normed fit index (NNFI) and comparative fit index (CFI), the threshold value for a good fit should be near 0.95. Finally, for the normed chi-squared statistic, the value should be less than 2 in order to indicate a good model fit [72,73].

### 4.3. Research Model I (TAM)

In Table 5, perceived ease of use (PEOU) significantly impacted perceived value (PV) (b = 0.420, *p* < 0.001). Both (PEOU) and (PV) (b = 0.649, *p* < 0.001) significantly impact continuance intention (CI) (b = 0.256, *p* = 0.001). Three research hypotheses are all supported. In addition, 26.2% of PV can be explained by the (PEOU) construct, and 62.2% of CI can be explained by (PEOU) and (PV) constructs.

### 4.4. Research Model II (TAM-IS Success Integrated Model)

#### 4.4.1. Analysis of Structural Model

Table 6 shows the path coefficients of the TAM-IS success integrated model. System quality (SYQ) has a significant impact on perceived ease of use (PEOU) (b = 0.727, *p* = 0.030). Perceived ease of use (PEOU) significantly affects perceived value (PV) (b = 0.209, *p* = 0.005). In addition, perceived value (PV) has a significant influence on continuance intention (CI) (b = 0.918, *p* < 0.001).

The results support the research question regarding the validity of the research model. System quality, information quality, and service quality can explain 31.5% of perceived ease of use. System quality, information quality, service quality, and perceived ease of use can explain 59.3% of perceived value. Perceived ease of use and perceived value can explain 78.3% of continuance intention.

#### 4.4.2. Analysis of Mediation Effects

This study used bootstrapping mediation analysis because it can provide confidential intervals to examine the indirect effects. Bias-corrected bootstrapping is one of the preferable bootstrapping mediation analysis methods [74,75]. The total effect of system quality (SYQ) to continuance intention (CI) (*p* > 0.05), information quality (INQ) to continuance intention CI (*p* > 0.05), and service quality (SEQ) to continuance intention CI (*p* > 0.05) were not supported, so it was not necessary to test the mediation effect.

### 4.5. Research Model III (TAM-ECM Integrated Model)

#### 4.5.1. Analysis of Structural Model

The path coefficients of the TAM-ECT model are shown in Table 7. Confirmation (EC) significantly impact both perceived value (PV) (b = 0.619, *p* < 0.001) and satisfaction (SAT) (b = 0.865, *p* < 0.001). Perceived ease of use (PEOU) (b = 0.173, *p* = 0.006), perceived value (PV) (b = 0.342, *p* = 0.009), and satisfaction (SAT) (b = 0.474, *p* < 0.001) all have significant effects on continuance intention (CI).

Perceived ease of use (PEOU) and confirmation (EC) could explain 63.9% of perceived value (PV). Confirmation (EC) can explain 78.2% of satisfaction (SAT). Perceived ease of use (PEOU), perceived value (PV), and satisfaction (SAT) can explain 73.9% of continuance intention (CI).

#### 4.5.2. Analysis of Mediation and Moderation Effects

In testing mediation effects, the total effect PEOU to CI, *p* < 0.05, the bias-corrected confidence interval does not include 0 (confidence interval of PEOU to CI = (0.041 0.377)). The existence of a total effect was supported. The total indirect effect PEOU to CI, *p* > 0.05, bias-corrected confidence interval does include 0 (confidence interval of PEOU to CI = (−0.004 0.138)). The existence of a total indirect effect was not supported. The total effect EC to CI, *p* < 0.05, bias-corrected confidence interval does not include 0 (confidence interval of EC to CI = (0.444 0.816)). The existence of a total effect was supported. The indirect effect EC → PV → CI, *p* < 0.05, bias-corrected confidence interval does not include 0 (confidence interval of EC → PV → CI = (0.041 0.461)). Thus, the hypothesis of the existence of an indirect effect was supported. The indirect effect EC → SAT → CI, *p* < 0.05, bias-corrected confidence interval does not include 0 (confidence interval of EC → SAT → CI= (0.191 0.683)). Thus, the hypothesis of the existence of an indirect effect was supported.

Habits (HAB) are a moderator in our proposed model. As shown in Table 8, the moderating effect of PV×HAB to CI is −0.309 (z = |−2.763| > 1.96, *p* = 0.006). As the value of *p* is less than 0.05, a moderating effect exists. The slope of PV to CI decreases by −0.309 units for every 1 unit of the moderator (HAB). SAT×HAB to CI is 0.179 (z =|1.919| < 1.96, *p* = 0.055). As *p* ≥ 0.05, a moderating effect does not exist.

## 5. Discussions

This study found that the TAM model has an explanatory power of 62.2%. By adding system quality, information quality, and service quality to the TAM model, the TAM-IS success integrated model has a better explanatory power of 78.3%. By increasing confirmation and satisfaction to the TAM model, the TAM-ECM model has an explanatory power of 73.9%, similar to that of the TAM-IS success integrated model. Because the explanatory power of the TAM-IS success integrated model is better than the TAM and the TAM-ECM, it can be inferred that the TAM-IS success integrated model has the best predictive ability for the continuance intention of using the agri-food traceability system. Since the three models have similar explanatory power, Taylor and Todd [76] suggested that the model with simplicity should be chosen for easy practical understanding. In this case, TAM is the most appropriate model in explaining the continuance intention of the agri-food traceability system because it uses only two predictors. Though the TAM-IS success integrated model has the best explanatory power (78.3%) with five independent variables, the TAM-ECM model has a similar explanatory power (73.9%) using only four predecessors.

Most of the proposed hypotheses are significant. The results of the supported hypotheses are summarized in Table 9.

Unsurprisingly, the results of the first proposed TAM model are consistent with recent IT studies [77,78,79,80,81,82]. All three hypotheses are supported. Perceived ease of use affects perceived value (H1.1), and both perceived ease of use (H1.2,) and perceived value (H1.3) have impacts on the continuance intention of the use of the agri-food traceability system.

Yet, not all hypotheses of the second proposed model (TAM-IS success integrated model) are supported. Among system quality, information quality, and service quality, only H2.1 (system quality has an impact on the perceived ease of use) is supported. The design quality of the system, no matter its interface or functions, plays a vital role in impacting users’ perception of ease of use or not (Shah and Attiq [83]). Hypotheses H2.7 (perceived ease of use has a positive and significant effect on perceived value) and H2.9 (perceived value has a positive and significant effect on continuance intention) are supported, as are H1.1 and H1.3. No mediation effect was found in the TAM-IS success integrated model.

The direct effects of the third proposed model (TAM-ECM integrated model) are all supported, except for the effect of perceived ease of use on perceived value (H3.3), which is close, but not significant (*p* = 0.068). This finding does not agree with the TAM assumption that perceived ease of use affects perceived usefulness (in this study, perceived value). The nonsignificant relationship between perceived ease of use and perceived value could be attributed to the cognition of the users of whether the value of the traceability system has met their expectation and confirmation level, which is much more important than the perceived ease of use of the system. In other words, the perceived value was directly positively related to the confirmation, but not to perceived ease of use. A confirmation that affects both perceived value (H3.1) and satisfaction (H3.2) could also prove that the effect of confirmation on the perceived value is stronger than that of perceived ease of use on perceived value. The surveyed farmers have at least one-year experience in using the agri-food traceability system. Their high degree of confirmation (average 5.6) means the performance of the system has met users’ expectations, and results in a high degree of perceived value (mean 5.68) and satisfaction (mean 5.72). Perceived ease of use (H3.4), perceived value (H3.5), and satisfaction (H3.6) influence continuance intention. Users’ perception of ease of use, value, and level of satisfaction affect their intention to continue using the agri-food traceability system. These findings are comparable to other IT studies [84,85,86].

Two mediating effects were found in the TAM-ECM integrated model. As confirmation is the predecessor of this model, users’ confirmation affects their continuance intention through either perceived value or satisfaction. In this study, users’ confirmation of their expectations would form a highly perceived value or satisfaction towards their continuance intention of traceability system usage. This phenomenon is justifiable in the expectation confirmation model, in which users’ satisfaction and reuse intention are determined by their initial expectation of the traceability system and the confirmation level. The mediating findings were also similar to Li [40] or Jin’s [44] study, in which perceived value or satisfaction mediate experience quality and behavioral intention in between.

In addition, to integrate TAM with ECM, this study integrated user habits in the third research model because habits help to explain users’ continuance intention to use the traceability system. Habit is a behavioral tendency resulting from previous experience [59]. Habits have a direct impact on purchase intention [57], or a mediating effect between satisfaction and repeated purchase intention [87]. This study takes a different viewpoint from Chiu et al. [62] and Hsu et al. [63] by considering habit as a moderator, as it is rarely found in the previous studies. This study revealed that user habits moderate the impact of perceived value on continuance intention (H3.8), but not on satisfaction and continuance intention (H3.9). This finding enriches the existing literature by acknowledging that habit has a stronger impact on the continuance intention for traceability system users with a high awareness of perceived value. Unlike the research of Khalifa and Liu [59], which showed that habits moderated the relationship between satisfaction and repurchase intention, this study reaches the same finding as of Hsu et al. [63]: the moderation effect of habits is insignificant. When the perceived value is taken into consideration, traceability system users may care more about obtaining benefits from the system, even though they might not be satisfied with the system in any way.

## 6. Theoretical Implications

The findings of this study provide several theoretical implications. First, this study proposed three models that are applicable in the context of agri-food traceability systems to explain users’ continuance intention by introducing TAM, TAM-IS success, and TAM-ECM integrated models from different theoretical perspectives. This approach is rarely found in IS studies on understanding users’ continuance intention of agri-food traceability system usage, especially in response to the COVID-19 pandemic.

Secondly, TAM concerns technology adoption; the IS success model focuses on the influences from the system, information, and service quality; whereas the ECM stresses the impacts from users’ confirmation level. This study integrated TAM with the IS success model, and TAM with ECM. This model integration approach gains valuable insights in understanding users’ continuance intention of agri-food traceability system usage.

Thirdly, this study proved that the TAM-IS success integrated model has the best explanatory power among the three models, showing that the integrated model provides a more thorough understanding of users’ continuance intention of an agri-food traceability system than either the TAM or IS success model alone.

Fourthly, the theoretical relevance of the mediating effects that either perceived value or satisfaction mediate confirmation and continuance intention, and the moderating effect that habits moderate the relationship between perceived value and continuance intention becomes evident. It is hoped that this study will contribute to the further advancement of the concept of system usage habits.

## 7. Practical Implications

The samples in this study demonstrated a high degree of confirmation on the agri-food traceability system. Users’ high confirmation level is the starting point that triggers the user’s continuance intention through mediators, perceived value, and satisfaction. To maintain, or even to raise, the positive confirmation level is mainly under the control of the traceability system provider. For example, the traceability system provider should continue to maintain or improve system and service quality, as in the TAM-IS success integrated model. This can help to increase users’ understanding of the system, and to enhance users’ experience when using it. For example, developers should ensure the availability of the traceability system. Whenever consumers scan the QR code on the agri-food, the system should be able to display the information promptly. The traceability system must consolidate with the logistics system. Developers should even improve the farmers’ transportation system, so the agri-food could be delivered to the market as soon as possible.

This study indicates that habits exert a significant moderating effect on the relationship between perceived value and continuance intention. This implies that habits are still the key factor in stimulating traceability system users’ continuance intentions. Accordingly, building users’ habits is an ultimate goal for traceability system providers. Without providing the users with perceived value, there is no point in instigating users’ habits. Moreover, this study reported that satisfaction and perceived value are the most important predictors of continuance intention. From the perspective of user management, a higher priority should be given to enhance users’ value recognition on the traceability system, and to improve their satisfaction. Thus, system providers could encourage frequent usage by providing incentives, and launching services sites locally to develop users’ habits.

This study has identified perceived value as the most significant factor in affecting users’ continuance intention in the TAM and TAM-IS success model, and satisfaction has the greatest influence on the continuance intention in the TAM-ECM. Namely, users who can appreciate the value of the traceability system, or are satisfied with the system, tend to have a greater tendency to use the system continuously. Consequently, traceability system providers should organize activities, such as conferences in this study, or events, to increase users’ understanding about this system. The more the users understand the benefits of using the traceability system, the more they will continue to use it. Exposing or introducing the traceability system to the public will attract more new users to use it. In this way, system developers could generate more revenue by recruiting new users, and using the extra income to improve the system performance, so that the users’ perceived value and satisfaction can be further enhanced.

## 8. Conclusions

In response to COVID-19, some studies proposed food safety solutions regarding food traceability systems, such as decentralized food supply chain management [88], digitalized meat supply chains [89], and future global traceability tools [90]. Although the technology acceptance model-information systems success model (TAM-IS) and expectation confirmation model (ECM) have been widely used in various fields, very few studies have combined the technology acceptance model-information systems success model (TAM-IS) and expectation confirmation model (ECM) together to explore users’ continuance intention. It is even rare to find a continuance intention study that introduced three comparative models under one research scenario. By integrating the technology acceptance model (TAM), the information systems success model (IS), and the expectation confirmation model (ECM), the novelty of this study is to develop three conceptual models explaining consumers’ continuance intention to use the traceability system. Also, this study investigated the relationships among three models, including constructs such as perceived ease of use, perceived value, system quality, information quality, service quality, confirmation, satisfaction, and continuance intention. The main contribution of the study is to enrich the scientific literature, and provide valuable insight to the food chain industry regarding consumers’ continuance intention to use the traceability system under the COVID-19 pandemic.

The paper has some limitations and future directions. Firstly, this study sampled traceability system users from a conference. The convenience sampling might result in data bias and distortions. Therefore, the inference of the study results was limited. Secondly, this research focused on China, which is an emerging economy. Future research could replicate this research, and make a comparison study in other countries. Thirdly, this research developed three models, and proposed a large number of hypotheses. Future research could adopt different theories to create a research model with a limited number of hypotheses to refine the insights. Finally, this study only investigated the user side of traceability systems; the factor of quality control might be a good start to understand the effects of human error and bias on the traceability systems on the manufacturer’s side.

## Figures and Tables

**Table 1:** Previous literature on traceability systems.

Author	Context	Theory Adopted	Dependent Variable
Li, Paudel, and Guo [20]	Vegetable traceability systems	Expanded TAM	Participation intention
Masudin, Ramadhani, Restuputri, and Amallynda [21]	Traceability system	Self-developed	Food cold chain performance
Yuan, Wang, and Yu [19]	Food traceability system	Customer value theory	Purchase intention
Pappa, Iliopoulos & Massouras [22]	Agri-food chains	TAM 2 and TPB	Intention to use
Chen, Wu, Pan, Siu, Gong, and Zhu [23]	Agricultural products traceability system	TAM	Intention to use
Abd Rahman, Singhry, Hanafiah, and Abdul [24]	Halal assurance system (HAS)	The resource-based view (RBV)	Readiness toward HAS
Kim and Woo [25]	Food traceability system	Extended TAM	Behavioral intention to use
Buaprommee and Polyorat [18]	Meat with traceability	Self-developed	Purchase intention
Chang, Tseng, and Chu [26]	Food traceability system	3M model of motivation and personality	Intention to purchase
Chen and Huang [17]	Food traceability system	Self-developed	Purchase intention
Al-Tal [27]	Food traceability systems	Information asymmetry	Willingness to pay
Heyder, Theuvsen, and Hollmann-Hespos [28]	Tracking and tracing systems	TAM2	Intention to use

**Table 2:** Results for the measurement model.

Constructs	Item	Significance of Estimated Parameters				Item Reliability		Construct Reliability	Convergence Validity
		Unstd.	S.E.	*t* Value	*p*	Std.	SMC	C.R	AVE
Perceived Ease of Use	PEOU1	1.000				0.717	0.514	0.863	0.616
	PEOU2	1.011	0.116	8.713	0.000	0.651	0.424		
	PEOU3	1.261	0.111	11.393	0.000	0.868	0.753		
	PEOU4	1.217	0.109	11.211	0.000	0.880	0.774		
Perceived Value	PV1	1.000				0.697	0.486	0.756	0.508
	PV2	0.921	0.109	8.472	0.000	0.685	0.469		
	PV5	0.938	0.114	8.198	0.000	0.755	0.570		
Confirmation	EDOC1	1.000				0.747	0.558	0.829	0.549
	EDOC2	0.892	0.095	9.374	0.000	0.702	0.493		
	EDOC3	0.918	0.091	10.101	0.000	0.760	0.578		
	EDOC4	0.961	0.095	10.088	0.000	0.753	0.567		
Satisfaction	SAT1	1.000				0.784	0.615	0.841	0.571
	SAT2	0.845	0.091	9.248	0.000	0.658	0.433		
	SAT3	0.967	0.091	10.673	0.000	0.744	0.554		
	SAT4	0.977	0.081	12.017	0.000	0.826	0.682		
Habit	UH1	1.000				0.843	0.711	0.874	0.698
	UH2	0.957	0.075	12.787	0.000	0.851	0.724		
	UH3	0.798	0.064	12.381	0.000	0.812	0.659		
Continuance Intention	CI1	1.000				0.827	0.684	0.773	0.536
	CI2	0.982	0.089	11.013	0.000	0.713	0.564		
	CI3	1.007	0.122	8.254	0.000	0.599	0.359		
System Quality	SYQ1	1.000				0.754	0.569	0.837	0.563
	SYQ2	0.945	0.097	9.780	0.000	0.710	0.504		
	SYQ3	1.131	0.105	10.742	0.000	0.768	0.590		
	SYQ4	1.145	0.110	10.412	0.000	0.768	0.590		
Information Quality	IQ1	1.000				0.778	0.618	0.793	0.561
	IQ3	0.913	0.087	10.444	0.000	0.733	0.537		
	IQ4	0.838	0.080	10.428	0.000	0.726	0.527		
Service Quality	SEQ1	1.000				0.738	0.545	0.806	0.513
	SEQ2	0.957	0.119	2.020	0.000	0.591	0.349		
	SEQ3	1.004	0.098	10.241	0.000	0.745	0.555		
	SEQ4	0.977	0.107	10.162	0.000	0.746	0.557		

Unstd.: unstandardized factor loadings; S.E.: standard error; Std: standardized factor loadings; SMC: square multiple correlations; CR: composite reliability; AVE: average variance extracted.

**Table 3:** Discriminant validity for the measurement model.

	AVE	Satisfaction	Service Quality	Information Quality	System Quality	Continuance Intention	Habits	Perceived Ease of Use	Perceived Value	Confirmation
Satisfaction	0.573	**0.757**								
Service Quality	0.513	0.326	**0.716**							
Information Quality	0.500	0.318	0.342	**0.707**						
System Quality	0.563	0.386	0.420	0.405	**0.750**					
continuance intention	0.540	0.370	0.331	0.341	0.391	**0.735**				
Habits	0.698	0.430	0.317	0.318	0.423	0.411	**0.835**			
Perceived Ease of Use	0.616	0.360	0.311	0.285	0.412	0.409	0.375	**0.785**		
Perceived Value	0.518	0.297	0.220	0.202	0.224	0.298	0.233	0.297	**0.720**	
Confirmation	0.549	0.369	0.314	0.302	0.371	0.333	0.348	0.306	0.270	**0.741**

Note: The items on the diagonal in bold represent the square roots of the AVE; off-diagonal elements are the correlation estimates.

**Table 4:** Model fit.

Model Fit Indicators	Criteria	TAM	TAM-IS	TAM-ECM
Normed Chi-squared	1 < χ^2^/DF < 3	1.564	1.697	1.673
RMSEA	<0.08	0.054	0.060	0.058
NNFI	>0.9	0.970	0.929	0.942
CFI	>0.9	0.978	0.941	0.952
GFI	>0.9	0.951	0.869	0.889
AGFI	>0.9	0.915	0.844	0.868
NFI	>0.9	0.943	0.877	0.879

**Table 5:** Regression coefficient of research model I (TAM).

DV	IV	Unstd	*p*-Value	Results
PV	PEOU	0.420	0.000	Supported
CI	PEOU	0.256	0.001	Supported
	PV	0.649	0.000	Supported

**Table 6:** Regression coefficient of research model II (TAM-IS Success Integrated Model).

DV	IV	Unstd	S.E.	Unstd./S.E.	*p*-Value	Std.	R^2^
PEOU	SYQ	0.727	0.335	2.171	0.030	0.617	0.315
	INQ	−0.229	0.405	−0.565	0.572	−0.203	
	SEQ	0.159	0.300	0.530	0.596	0.141	
PV	SYQ	−0.217	0.256	−0.849	0.396	−0.249	0.593
	INQ	0.544	0.304	1.793	0.073	0.652	
	SEQ	0.154	0.216	0.716	0.474	0.185	
	PEOU	0.209	0.074	2.818	0.005	0.282	
CI	PEOU	0.161	0.087	1.861	0.063	0.183	0.783
	PV	0.918	0.169	5.444	0.000	0.772	

**Table 7:** Regression coefficient of research model III (TAM-ECM Integrated Model).

DV	IV	Unstd	S.E.	Unstd./S.E.	*p*-Value	Std.	R^2^
PV	PEOU	0.118	0.065	1.825	0.068	0.151	0.639
	EC	0.619	0.093	6.626	0.000	0.711	
SAT	EC	0.865	0.091	9.500	0.000	0.884	0.782
CI	PEOU	0.173	0.063	2.731	0.006	0.202	0.739
	PV	0.342	0.131	2.619	0.009	0.312	
	SAT	0.474	0.107	4.424	0.000	0.485	

**Table 8:** Moderator Effects.

DV	IV	Estimate	S.E.	Z-Value	*p*-Value
CI	PEOU	0.149	0.090	1.655	0.098
	PV	0.413	0.151	2.740	0.006
	SAT	0.195	0.173	1.128	0.259
	HAB	0.215	0.090	2.398	0.016
	PV×HAB	−0.309	0.112	−2.763	0.006
	SAT×HAB	0.179	0.093	1.919	0.055

**Table 9:** Summary of the supported hypotheses.

Proposed Model	Hypothesis
TAM	H1.1. Perceived ease of use has a positive impact on perceived value H1.2. Perceived ease of use has a positive impact on behavioral intention H1.3. Perceived value has a positive impact on behavioral intention
TAM-IS	H2.1. System quality has a positive and significant effect on perceived ease of use H2.7. Perceived ease of use has a positive and significant effect on perceived value H2.9. Perceived value has a positive and significant effect on continuance intention H2.10. There are mediation effects in this model
TAM-ECM	H3.1. Confirmation has a positive and significant effect on perceived value H3.2. Confirmation has a positive and significant effect on satisfaction H3.4. Perceived ease of use has a positive and significant effect on continuance intention H3.5. Perceived value has a positive and significant effect on continuance intention H3.6. Satisfaction has a positive and significant effect on continuance intention H3.7. There are mediation effects in this model H3.8. User habits have a moderator effect on the relationship between perceived value and continuance intention

## Data Availability

The data presented in this study are available on request from the corresponding author.

## Appendix A

Perceived Ease of Use
1. I can easily learn how to use the agri-food tracing system
2. It does not take me much time to learn how to use the agri-food traceability system
3. It is easy for me to use the agri-food traceability system
4. In general, I do not think it is difficult for me to use the agri-food traceability system
Perceived Value
1. The quality of agri-food is guaranteed by using the agri-food tracing system
2. The use of the agri-food traceability system makes the management of agri-food standardized
3. The use of the agri-food tracing system makes the price of agri-food higher
4. The use of the agri-food tracing system makes the income of agri-food higher
5. In general, the use of the agri-food tracing system is useful to me
Confirmation
1. The gain from using the agri-food traceability system is greater than I expected
2. The functions provided by the agri-food tracing system are more than I expected
3. The functions provided by the agri-food tracking system are better than I expected
4. In general, all my expectations of the agri-food traceability system have been met after using it
Satisfaction
1. I am satisfied with the training and service of the agri-food traceability system
2. I am satisfied with the experience of using the agri-food traceability system
3. I am satisfied with the effect of using the agri-food traceability system
4. In general, I am satisfied with the agri-food traceability system
Habits
1. It has become my habit to use the agri-food traceability system
2. Using the agri-food traceability system is a natural thing for me
3. When I manage the sale of agri-food, the agri-food traceability system was an obvious choice for me
Continuance Intention
1. I would like to continue using the agri-food tracing system
2. I will continue to use the agri-food traceability system
3. I will recommend the agri-food traceability system to other farmers
System Quality
1. The interface of the agri-food tracing system is very friendly
2. The security of the agri-food tracing system is very good
3. The stability of the agri-food tracing system is very good
4. The maintenance service of the agri-food tracing system is very good
Information Quality
1. The information provided by the agri-food tracing system is complete
2. The information provided by the agri-food tracing system is new, and updated in time
3. The information provided by the agri-food tracing system is reliable
4. The information provided by the agri-food tracing system is rich in content
Service Quality
1. The provider of the agri-food traceability system has the ability to solve the user’s questions
2. The provider of the agri-food tracing system puts the interests of farmers first
3. The provider of the agri-food traceability system will solve the problems in time
4. The service of the provider of the agri-food traceability system is continuous and stable
